# Superior Potency of Synthetic Virus-like Structures in Vaccine-Induced Antibody Responses Compared to Qβ Bacteriophage Virus-like Particles

**DOI:** 10.3390/v17040579

**Published:** 2025-04-17

**Authors:** Alexander R. Meyer, Libo Li, Wei-Yun Wholey, Bryce Chackerian, Wei Cheng

**Affiliations:** 1 Department of Pharmaceutical Sciences, University of Michigan, 428 Church Street, Ann Arbor, MI 48109, USA; meyerar@umich.edu (A.R.M.); libol@umich.edu (L.L.); weiyun@umich.edu (W.-Y.W.); 2 Department of Molecular Genetics and Microbiology, School of Medicine, University of New Mexico, Albuquerque, NM 87131, USA; bchackerian@salud.unm.edu; 3 Department of Biological Chemistry, University of Michigan Medical School, 1150 W. Medical Center Dr., Ann Arbor, MI 48109, USA

**Keywords:** synthetic virus-like structures, Qβ bacteriophage, virus-like particles, antibody, affinity maturation

## Abstract

Virus-like particles are a well-established platform for vaccines, although the molecular mechanisms that underlie the extraordinary potency of many virus-like particles in eliciting strong antibody responses remain incompletely understood. Here, we show that synthetic virus-like structures, a new platform that we have recently developed, are superior to bacteriophage Qβ-based virus-like particles for the induction of long-term neutralizing antibody responses. For the same antigen, both platforms induced antibodies with comparable affinities. The resulting antigen-specific antibodies had similar binding on-rates and off-rates. However, synthetic virus-like structures induced a much higher concentration of functional antibodies in the serum than Qβ-based virus-like particles, suggesting that synthetic virus-like structures are more potent than Qβ-based virus-like particles in the induction of long-lived plasma cells.

## 1. Introduction

Virus-like particles (VLPs) are a well-established form of vaccine, exemplified by the FDA-approved human papillomavirus vaccines [1]. The characteristic feature of VLPs is the multivalent and oriented display of antigens (Ags) in a relatively high spatial density on submicron-sized structures [2], similar to the surface of many eukaryotic viruses. This high-density display of Ags is important for the immunogenicity of these particles [3,4,5,6], although the underlying mechanisms of B cell activation by these Ags remain incompletely understood. Among different VLPs that have been reported in the literature, bacteriophage Qβ-derived VLPs [7] have emerged as a platform for vaccine development [8,9] and also as a model system for the mechanistic understanding of Ab responses to virus-like immunogens, especially during the early phases of Ab responses [5,10,11,12]. However, Qβ VLPs are mixtures of phage proteins and single-stranded RNA self-assembled from *Escherichia coli* cell culture in diverse forms [13]. The different viral protein components and the under-defined nucleic acids in these structures render it challenging to dissect the molecular basis for the immunogenicity of these particles. In an effort to understand the mechanisms of B cell responses to virus-like immunogens, we have recently developed a reductionist system of synthetic virus-like structures (SVLS) that are based on unilamellar liposomes. The diameters of SVLS are around 120 nm [14,15,16], which is very close to the size of enveloped viruses such as HIV-1, influenza virus, and SARS-CoV-2 [17,18,19]. SVLS are assembled completely in vitro using highly purified individual components, including proteins, lipids, and nucleic acids. We have developed and demonstrated site-specific conjugation of protein Ags of interest onto liposomes, which is key in the orientation-specific display of epitopes on these structures [15,16]. SVLS are highly modular supramolecular structures both in the density of protein Ag display and in the internal contents that they encapsulate [14,15,16,20,21]. The defined compositions, as well as the modular nature of these structures, have allowed us to unravel the mechanisms of B cell responses towards virus-like immunogens in ways that were not possible before [14,20,21]. In particular, we have reported recently that the system of SVLS rivals Qβ-derived VLPs in early Ab responses induced in mice, which occurred within 11 days after a single immunization of respective immunogens in the absence of additional adjuvants [14]. Here, we further characterize the long-term Ab responses and compare them between these two different forms of multivalent Ags. We show that SVLS are much more potent than Qβ-derived VLPs in the induction of long-term Ab responses in mice.

## 2. Materials and Methods

### 2.1. Synthesis of SVLS

**Figure 1:**
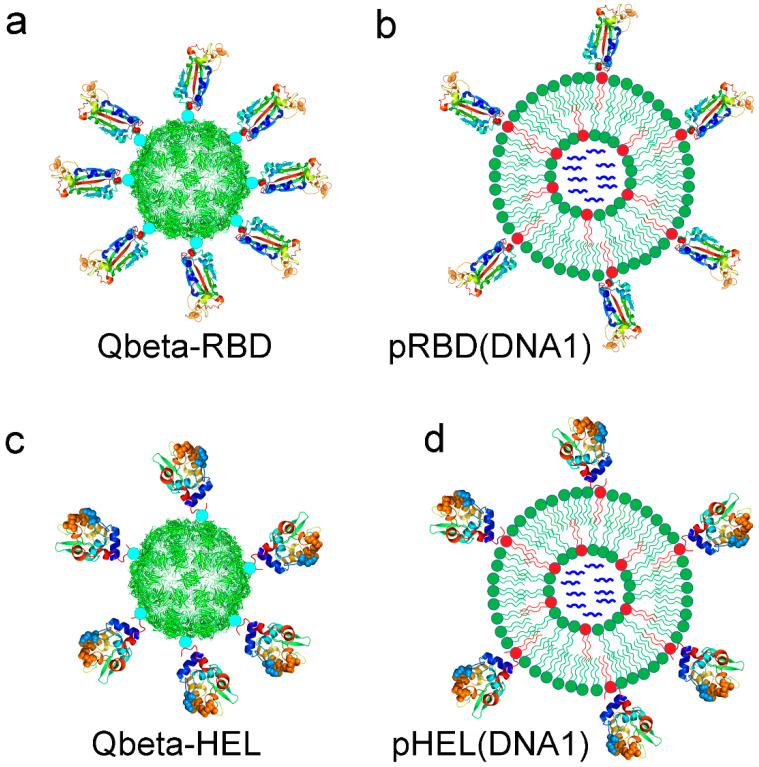
Schematic of four virus-like immunogens used in the current study. (**a**,**c**) are bacteriophage Qβ VLPs conjugated with RBD as Qbeta-RBD in (**a**) and with HEL as Qbeta-HEL in (**c**), respectively. (**b**,**d**) are SVLS conjugated with RBD as pRBD(DNA1) in (**b**) and with HEL as pHEL(DNA1) in (**d**), respectively. The bacteriophage Qβ VLP is represented by the backbone model for phage Qβ capsid in PDB 5vlz [28]. The ribbon diagram of RBD used the RBD structure in PDB 6m0j [29], and the ribbon diagram of HEL used the HEL structure in PDB 1c08 [30]. The SVLS use unilamellar liposomes as the backbones. The internal space of the liposome is loaded with single-stranded DNA oligo DNA1 to mimic the nucleic acid genomes inside typical biological viruses. Throughout, all particle surfaces were conjugated with respective protein Ags site-specifically via maleimide chemistry with defined epitope densities. Specifically, Qbeta-RBD has 200 molecules of RBD per particle; pRBD(DNA1) has 46 molecules of RBD per structure; Qbeta-HEL has 10 molecules of HEL per particle; and pHEL(DNA1) has 9 molecules of HEL per structure. The cyan circles in (**a**,**c**) represent the heterobifunctional crosslinker SMPH. Proteins and their respective spherical structural backbones are not drawn to scale.

All SVLS used in this study were prepared following protocols we published previously [14,15,16,21]. These SVLS were constructed using nonimmunogenic lipids, with phosphatidylcholine and cholesterol comprising ≥ 99% of all lipids. These structures display protein antigens (Ags) of choice in a specific orientation on their surfaces with a regulated epitope density (ED) that quantitatively mimics the surface of naturally occurring viruses [22,23] (Figure 1). The HEL recombinant protein used in this study contained two site-directed mutations, R73E and D101R, and was overexpressed in *E. coli* and purified to >95% purity following our established protocols [15]. Similarly, the SARS-CoV-2 RBD recombinant protein used in this study was overexpressed using 293F cells and purified to >95% purity following our established protocols [16]. DNA1 was custom-synthesized by IDT. The sequence of DNA1 is as follows: 5′-TCCATGACGTTCCTGACGTT-3′. ED was quantified using methods that we established previously [15,16,24,25] that were also validated by single-molecule fluorescence techniques, which were developed and established in-house [14,26,27]. For SVLS with internal nucleic acids (iNAs), the average number of iNA molecules per SVLS was also quantified using methods that we established previously [15,16]. Particle size was measured using a Malvern Zetasizer Nano ZSP at 20 °C.

### 2.2. Conjugation of RBD or HEL to Bacteriophage Qβ Virus-like Particles (VLPs)

The bacteriophage Qβ VLPs were prepared as described [9]. The purified RBD or HEL recombinant proteins, which each contained a single reactive cysteine near their respective C-terminus, were conjugated to the surface of the VLPs using the heterobifunctional crosslinker Succinimidyl-6-[(β-maleimidopropionamido)hexanoate] (SMPH). Briefly, the VLPs were first derivatized with SMPH at a 10-fold molar excess of SMPH over Qβ coat protein. The mixture was incubated at 22 °C for two hours, and the excess crosslinker was removed by centrifugation at 4 °C in an Amicon Ultra-4 centrifugal unit with a 100 kD cutoff. The RBD or HEL protein was then added to the derivatized VLPs, and the mixture was incubated at 4 °C overnight for RBD or at 20 °C for 1 h for HEL. Free proteins were then removed by centrifugation at 4 °C in an Amicon Ultra-4 centrifugal unit with a 100 kD cutoff. The quantity of conjugated proteins was assessed using a denaturing polyacrylamide gel based on the intensity from silver staining compared to a standard curve obtained from the same gel.

### 2.3. Mice Immunizations

All animal procedures were approved by the University of Michigan Animal Care and Use Committee. Female C57BL/6 or BALBc/J mice (8 weeks, Jackson Laboratory, Bar Harbor, ME, USA) were used for immunizations. Prior to inoculation, all injection samples were filtered through 0.45 µm pore size membrane. Then, 100 µL samples at a dose between 0.1 and 0.3 µg of respective protein Ags were injected into each mouse subcutaneously, 50 µL in each flank. Throughout the study, no adjuvants other than the SVLS or Qβ-conjugated structures were administered, and only a single injection was administered. Mouse blood was collected submentally using Microvette serum collection tubes (Sarstedt, Nümbrecht, Germany) three days before the first injection and on the following days post immunization over the course of approximately one year: 5, 11, 25, 39, 67, 95, 123, 151, 179, 207, 235, 263, 291, 319, and 347. The sera were harvested by centrifugation at 10,000× *g* for 5 min, aliquoted, and immediately frozen and stored at −80 °C.

### 2.4. Enzyme-Linked Immunosorbent Assay (ELISA)

Blood serum was tested by ELISA to quantitate Ag-specific IgG responses to various immunizations. To this end, 96-well plates (Nunc MaxiSorp, Invitrogen, Waltham, MA, USA) were coated overnight at 4 °C with either 200 ng of soluble HEL (sHEL) or 320 ng of soluble RBD (sRBD) per well in PBS. After blocking with 1% Bovine Serum Albumin (BSA, Thermo Fisher Scientific, Waltham, MA, USA) in PBS, mouse sera of specified dilution factors were added to each well for incubation at 22 °C for 2 h. After three washes using PBS with 0.05% Tween 20, secondary goat anti-mouse IgG Fc-HRP antibody (# 1033-05, SouthernBiotech, Birmingham, AL, USA) was added in blocking buffer at 1:6000 dilution and incubated for 1 h at 22 °C. Following three washes, 100 µL of substrate 3,3′,5,5′-Tetramethylbenzidine (Thermo Fisher Scientific, Waltham, MA, USA) was added to each well and incubated in the dark for 10 min. The reaction was stopped by the addition of 100 µL 2M sulfuric acid in each well. The optical density of each well at 450 nm was measured using a microplate reader (Bio-Tek Synergy HT, Winooski, VT, USA). All the OD values reported were background subtracted by comparing two wells coated with soluble protein and PBS, respectively. To estimate RBD-specific IgG concentration in mouse sera, we used a commercial RBD-specific monoclonal mouse IgG2a (mAb1, BioLegend, San Diego, CA, USA, CAT#944803) as the known reference to construct standard curves for interpolation of RBD-specific IgG concentration. To estimate HEL-specific IgG concentration in mouse sera, we used a HEL-specific monoclonal mouse IgG1 (mAb2, clone HyHEL10, a special gift from Dr. Irina Grigorova at the University of Michigan Ann Arbor) as the known reference to construct standard curves for interpolation of HEL-specific IgG. Before interpolation, all sera samples were properly diluted to reach final OD values between 0.2 and 0.5.

### 2.5. Preparation of HIV-1 Virions Pseudotyped with SARS-CoV-2 Envelope

The HIV-1 pseudotyped with SARS-CoV-2 envelope was prepared following the house-published protocols [21] but with important modifications. Specifically, we have replaced the firefly luciferase gene in the provirus-containing plasmid pNL4-3 luc R-E- [31] (ARP-3418, NIH AIDS Research and Reference Reagent Program, Germantown, MD, USA) with a codon-optimized Nanoluc luciferase [32] (GenBank: AFI79290.1) in-house, which has allowed us to use much less sera and fewer host cells to conduct a highly sensitive neutralization assay and determine the titer of sera neutralization. Briefly, HEK 293T/17 cells (ATCC, Manassas, VA, USA) were cultured at 37 °C with 5% CO_2_ in DMEM supplemented with 10% FBS (HyClone Laboratories, Logan, UT, USA). Moreover, 10^6^ 293T cells in a 2 mL culture volume were seeded overnight in a 35 mm dish before transfection using the TransIT LT-1 transfection reagent (Mirus Bio, Madison, WI, USA). For each dish, 2 µg of the provirus-containing plasmid pNL4-3 Nanoluc R^-^E^-^ was used to make the transfection reagent mixture, together with 1 µg of envelope expression plasmid pcDNA3.1 SARS-CoV-2 S D614G [33]. The plasmid pcDNA3.1 SARS-CoV-2 S D614G was a gift from Jeremy Luban (Addgene plasmid # 158075; http://n2t.net/addgene:158075; RRID:Addgene_158075; accessed on 25 January 2021). The RBD amino acid sequence 328-537 encoded in this plasmid is identical to the sequence of RBD that we studied here. The transfection reagent mixture was incubated at room temperature for 15 min before drop-wise addition to the culture medium, as we had performed previously [34]. At 6 h post transfection, the culture medium, together with the transfection reagents, was replaced with fresh complete medium, and the incubation was continued at 37 °C with 5% CO_2_. At 48 h post transfection, the entire culture medium containing single-cycle HIV-1 viruses was collected and filtered through a 0.45 µm syringe filter (Millex-HV PVDF, Merck Millipore Ltd., Tullagreen, Carrigtwohill, Co. Cork, Ireland). The filtrate was then aliquoted on ice, flash-frozen in liquid nitrogen and stored in a −80 °C freezer. The concentration of virion particles was quantitated using a HIV-1 p24 ELISA kit (CAT#XB-1000, XpressBio, Frederick, MD, USA) as we described previously [34].

### 2.6. Virion Neutralization Assay

The virion neutralization assay follows the protocols we established previously [34] but with important modifications to determine the neutralization titer. HIV-1 virions pseudotyped with SARS-CoV-2 envelope containing 3.7 ng of HIV-1 p24 were incubated with mouse sera at varied dilutions at 37 °C for one hour and then layered onto Huh-7.5 cells to initiate infection at 37 °C for 2 h. At the end of 2 h, fresh medium was added to each well in a 96-well plate, and the incubation was continued at 37 °C with 5% CO_2_. Nanoluc luciferase activity was measured 48 h after infection. Briefly, the culture medium was removed, and the cells were rinsed with 200 µL of warm DPBS. Next, 110 µL of Nano-Glo luciferase reagent (CAT#N1120, Promega, Madison, WI, USA) that was just warmed to room temperature was freshly diluted and added to each well. The cells were incubated for three minutes at room temperature to allow cell lysis. After three minutes, 100 µL of lysate from each well was transferred to a single well in a 96-well black microtiter plate (Costar). Luminescence was measured using a Synergy^TM^ HT multi-mode plate reader (BioTek Instruments Inc., Winooski, VT, USA), and the background luminescence was subtracted using Huh-7.5 cells without virus infection. The luminescence readings for cells incubated with viruses without animal sera were set as 100% infectivity. The luminescence readings from other wells were all normalized based on this and plotted as relative infectivity. The relative infectivity as a function of the sera dilution factor was fitted to a sigmoid function [27,35] with top and bottom plateaus constrained to 100 and 0%. The neutralization titer of a serum sample is expressed as the serum dilution (the dilution before adding to cells) required to reduce the relative infectivity by 50%.

### 2.7. BioLayer Interferometry Measurements

Affinity maturation of Ag-specific antibodies in animal sera, together with binding and dissociation rate constants, were measured using BioLayer interferometry. Briefly, the streptavidin sensor (Sartorius Corp, Göttingen, Germany) was coated with 50 nM RBD or HEL in 1× PBS for 30 min at 20 °C, which was followed by washing in PBS for 10 min. For this application, both RBD and HEL were site-specifically biotinylated in-house using EZ-link maleimide-PEG2-biotin (ThermoFisher, Waltham, MA, USA, CAT#A39261). After sensor loading, the sensors were dipped into sera of varied dilutions in Buffer 1 (1× PBS with 0.05% Tween 20 and 0.1% BSA) to measure the binding in real time using an OctetRed BioLayer Interferometer (ForteBio, Fremont, CA, USA) equipped with 8 sensor positions that were read simultaneously. The binding measurement was continued for the indicated time, which was followed by dipping the sensors into Buffer 1 for the indicated time to monitor the dissociation in real time. The measurements were performed at 20 °C throughout. A PBS-coated sensor control was included and subtracted from all the kinetic data before quantitative analysis. The indicator of affinity maturation, I_am_, was determined by using the amplitude information from both binding and dissociation curves, specifically as I_am_ = (A1 + A2)/A1 × 100%, in which A1, a positive number, is the final amplitude at the end of binding measurement, and A2, a negative number as the result of dissociation, is the final amplitude at the end of dissociation measurement, as we illustrated previously [21]. In the current study, most binding and dissociation events were measured continuously in real time for 50 min each. For experiments to determine the rate of serum IgG binding to HEL, the binding and dissociation were monitored continuously for 6000 s for a better definition of the rate constants.

### 2.8. Measurement of Ag-Specific IgG Concentration in Animal Sera

We measured the concentration of Ag-specific IgG in the sera of immunized animals using streptavidin-coated magnetic beads. Specifically, high-capacity Magne streptavidin beads (Promega, Madison, WI, USA, CAT#V7820) were first exchanged into PBS buffer and then mixed with biotinylated RBD or HEL for binding at 20 °C for 1 h with constant mixing on a lab vortexer. After this, the beads were washed 3 times using Buffer 1 and then mixed with sera also diluted in Buffer 1. The binding was continued at 20 °C for 2 h with constant mixing. Finally, the supernatant was removed, and the bound IgG on beads was resuspended in nonreducing Laemmli sample buffer at 95 °C for 10 min. The amount of Ag-specific IgG was then quantitated by running a Western blot using mouse IgG (BioLegend, San Diego, CA, USA, CAT#944803) of known mass as a standard. The secondary antibody used was goat anti-mouse IgG (Fc specific) conjugated with alkaline phosphatase from Sigma-Aldrich (Sigma-Aldrich, St. Louis, MO, USA, CAT#A1418) and used at 1:3000 dilution.

## 3. Statistical Analysis

Statistical analysis was carried out using the Statistics toolbox in MATLAB R2023a (MathWorks, Natick, MA, USA) or the statistical analysis tools in OriginLab (Northampton, MA, USA). A comparison of two independent variables was carried out using a two-sample *t*-test. Data sets with more than two independent variables were analyzed using a one-way analysis of variance as indicated. *p*-values less than 0.05 were considered statistically significant. The details of all statistical analyses are described explicitly throughout the paper. All data points in the figures were reported as mean ± standard error unless otherwise noted.

## 4. Results

For comparison between SVLS and Qβ VLPs, we have chosen two protein Ags for conjugation onto the surface of these structures for immunization studies. One is the purified receptor-binding domain (RBD) of SARS-CoV-2, the causative agent of the COVID-19 pandemic [36]. The second is a purified mutant version of hen egg lysozyme (HEL), a well-characterized protein Ag that has been used extensively in immunological studies [37]. RBD and HEL are not related to each other in evolution, nor do they possess any significant sequence or structural similarities. Therefore, similar observations made for both protein Ags may suggest general relevance. Each protein was displayed in a specific orientation with a defined ED on the surface of the respective structures, as schematically shown in Figure 1.

**Figure 2:**
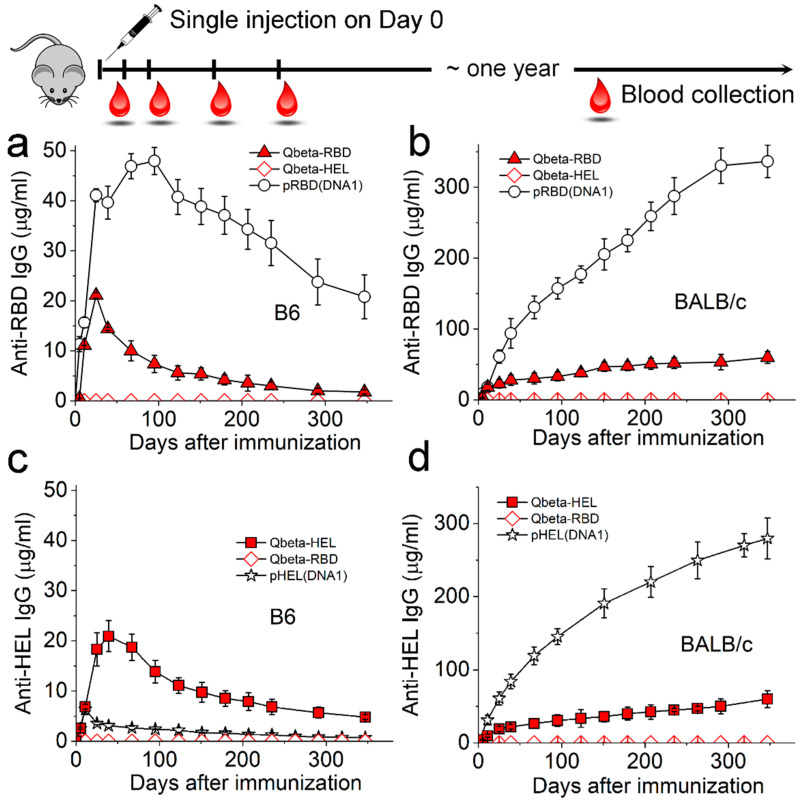
Duration of the IgG response induced by Qβ- or SVLS-conjugated Ags. A single injection followed by blood collection is schematically shown on top. (**a**,**b**) show concentrations of RBD-specific IgG in mouse sera from B6 (**a**) or BALB/c mice (**b**) collected over 300 days after a single injection of various agents listed in each inset. All IgG concentrations were measured using ELISA based on standard curves obtained from a reference monoclonal IgG mAb1 (Section 2.4). The doses of RBD on each particle were 0.24 µg per animal. (**c**,**d**) show concentrations of HEL-specific IgG in mouse sera from B6 (**c**) or BALB/c mice (**d**) collected over 300 days after a single injection of various agents listed in each inset. All IgG concentrations were measured using ELISA based on standard curves obtained from a reference monoclonal IgG mAb2 (Section 2.4). The doses of HEL on Qβ-HEL were 0.3 µg per animal, and the doses of HEL on pHEL(DNA1) were 0.1 µg per animal. Throughout Figure 2, N = 4 (4 animals per group) for each time point.

We found that a single injection of each particulate Ag at a submicrogram Ag dose was sufficient to elicit long-lived Ag-specific IgG responses in both C57BL/6 (B6) (Figure 2a,c) and BALB/c mice (Figure 2b,d) over the time span of one year, as measured by Ag-specific ELISA. This is consistent with what we reported recently, in which a multivalent Ag display on a virion-sized structure alone can seed long-lived Ab responses in mice [21]. However, we noted that the Ag-specific IgG induced by the SVLS platform was much stronger than that induced by the Qβ platform for the same Ags (Figure 2a,b,d), with the only exception of pHEL(DNA1) in B6 mice (Figure 2c). Three weeks after immunization, the anti-RBD IgG induced by Qβ-RBD in B6 mice (red triangles) was overall much less than that induced by pRBD(DNA1) (circles). The diamonds in Figure 2a represent the control sera from B6 mice immunized with an irrelevant Ag Qβ-HEL. This control demonstrates that the IgG responses are specific towards the protein Ags displayed on the surface of these structures. Similarly, as shown in Figure 2b, the anti-RBD IgG induced by Qβ-RBD (red triangles) in BALB/c mice was three-fold less than that induced by pRBD(DNA1) (circles) three weeks after immunization. This difference became even more pronounced as mice grew older during the year. As shown in Figure 2d, a much stronger Ab response induced by pHEL(DNA1) (stars) was also observed in BALB/c mice than that induced by Qβ-HEL at the same ED (red squares). In this group of experiments, the only exception is the anti-HEL IgG induced by Qβ-HEL in B6 mice (Figure 2c, red squares), which was substantially higher than that induced by pHEL(DNA1) (Figure 2c, stars). As we know, HEL is the only protein Ag in pHEL(DNA1), and HEL by itself is insufficient to elicit cognate T cell help in mice from the B6 genetic background [38]. In contrast, the structural proteins in Qβ VLPs are known to recruit potent T cell help in B6 mice and, therefore, enhance Ab responses towards the surface Ag, as we showed previously [25]. In summary, both Qβ and SVLS platforms induced durable Ab responses in two different strains of lab mice from a primary immune response. However, long-term Ab responses induced by SVLS were much stronger than those induced by Qβ conjugates in the presence of T cell help.

**Figure 3:**
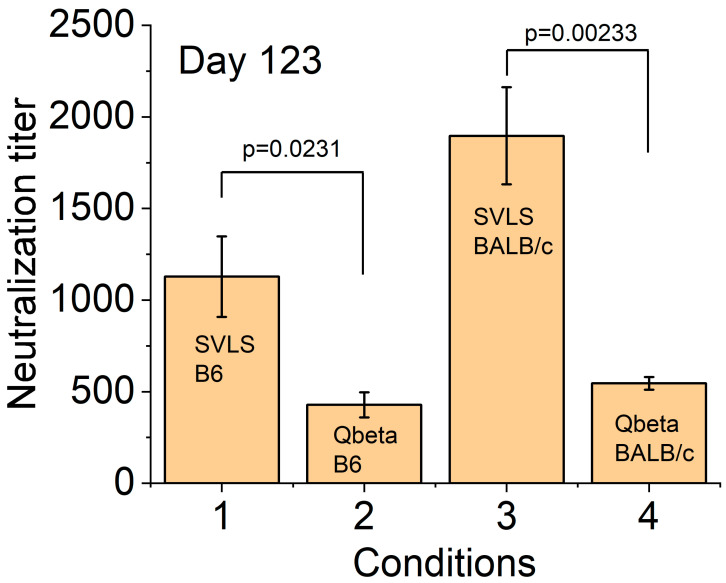
Titers of pseudovirion neutralization as determined for mice sera collected on Day 123 post immunization with respective particulate Ags. For Conditions 1 and 3, the SVLS is pRBD(DNA1). For Conditions 2 and 4, the Qβ is conjugated with RBD. N = 4 (4 animals per group) for each condition.

The choice of RBD as the protein Ag conjugated on these particles allows us to assess the functionality of the RBD-specific IgG using a pseudovirion neutralization assay that has been well established in the literature and also in our lab [14,16,21]. We prepared HIV-1-based pseudovirions that displayed the full-length cognate S protein of SARS-CoV-2 (Section 2.5). This pseudovirion-based reporter system has been rigorously compared with others and validated as an effective approach for the quantitative assessment of serological immunity against SARS-CoV-2 [39]. We determined the pseudovirion neutralization titer for the animal sera that were collected on Day 123 after respective immunizations. As shown in Figure 3, the sera collected from B6 mice immunized with a single dose of pRBD(DNA1) (Condition 1) yielded a neutralization titer of 1130 ± 220. This titer is 2.6-fold higher than that of B6 mice after a single immunization of Qβ-RBD at the same Ag dose (Condition 2), with a *p*-value of 0.0231 from a one-way ANOVA test. Similarly, the sera collected from BALB/c mice immunized with a single dose of pRBD(DNA1) produced a pseudovirion neutralization titer of 1900 ± 260 (Condition 3). This value is 3.5-fold higher than that of BALB/c mice after a single immunization of Qβ-RBD (Condition 4), with a *p*-value of 0.00233 from a one-way ANOVA test. These data show that the higher levels of RBD-specific IgG induced by SVLS as measured by ELISA in both B6 and BALB/c mice are correlated with higher levels of neutralization titer observed in SARS-CoV-2 pseudovirion infection in cell culture.

Why do the RBD-specific IgGs induced by Qβ-RBD have lower neutralization potency than those induced by SVLS? While the physical concentration of the IgG could be a contributing factor, as suggested by ELISA, it is also important to consider the affinity of the IgG towards its target. Instead of differences in their concentrations, could these IgGs have lower affinity to their target? This question cannot be addressed by ELISA, as we have seen from our recent studies [21]. The IgG concentration measured by ELISA using reference Abs is likely to underestimate the true concentrations of Ag-specific IgG because Ab affinities can change with time due to affinity maturation induced by immunization. To examine if this lower potency in pseudovirion neutralization is due to a potential defect in antibody affinity after immunization, we have thus measured Ab affinity maturation directly using BioLayer interferometry.

**Figure 4:**
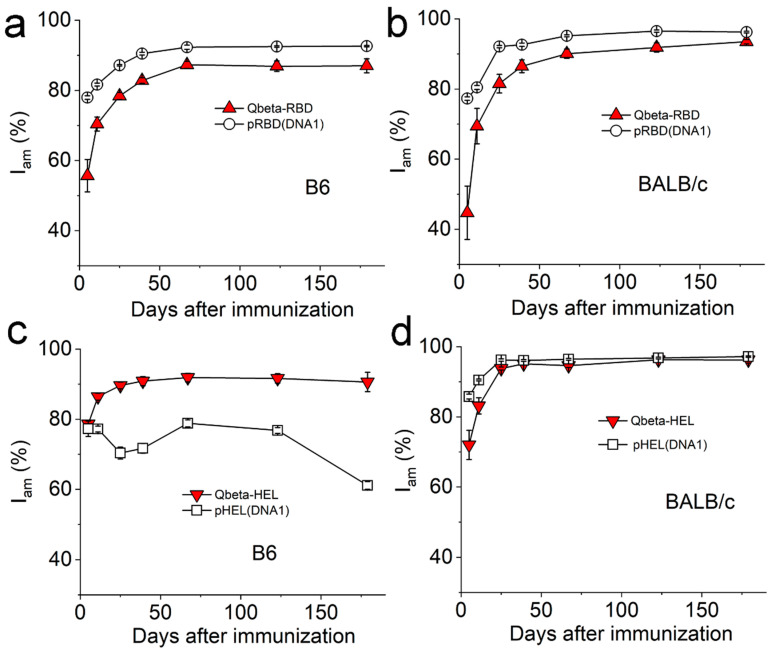
The quantitative progress of Ag-specific Ab affinity maturation in mice immunized with various agents. (**a**,**b**) show I_am_ of RBD-specific IgG in mouse sera from B6 (**a**) or BALB/c mice (**b**) collected over 6 months after a single injection of various agents listed in each inset. The doses of RBD on each particle were 0.24 µg per animal. (**c**,**d**) show I_am_ of HEL-specific IgG in mouse sera from B6 (**c**) or BALB/c mice (**d**) collected over 6 months after a single injection of various agents listed in each inset. The doses of HEL on Qβ-HEL were 0.3 µg per animal, and the doses of HEL on pHEL(DNA1) were 0.1 µg per animal. Throughout this figure, I_am_ was calculated directly from binding and dissociation traces measured using BioLayer interferometry (Section 2.7); N = 4 (4 animals per group) for each time point.

We quantify the process of Ab affinity maturation by measuring the indicator of affinity maturation, I_am_, as we published recently [21]. Briefly, I_am_ quantifies the affinity of an Ab relative to a perfect Ab that does not dissociate upon binding to its target (off rate = 0 s^−1^). Therefore, the experimental value of I_am_ is a number between 0 and 100%, with 100% being a perfect Ab. As Ab affinity improves with time due to affinity maturation, the value of I_am_ is expected to increase towards 100%. This was exactly what we observed after all the immunizations we conducted, except pHEL(DNA1) immunization in B6 mice. As shown in Figure 4, with the exception of pHEL(DNA1) immunization in B6 mice (Figure 4c squares), affinity maturation was observed following all the immunizations in both B6 and BALB/c mice using these particulate Ags, indicated by the fast rise of I_am_ within the first month after immunization which then approached a plateau around two months after immunization. Although these traces of I_am_ started from different values, an indication of differing Ab affinities on Day 5 at the beginning of immune responses, eventually, they all approached 90–95% relative to a perfect Ab. Notably, in the absence of cognate T cell help, there was no improvement in Ab affinity during this period of time, as shown in Figure 4c for pHEL(DNA1) immunization in B6 mice (squares), similar to what we showed previously for pHEL(DNA1) immunization in T cell receptor-deficient mice [21]. These results showed that the weaker neutralizing Ab responses induced by Qβ-conjugated particles are not due to defects in affinity maturation.

**Figure 5:**
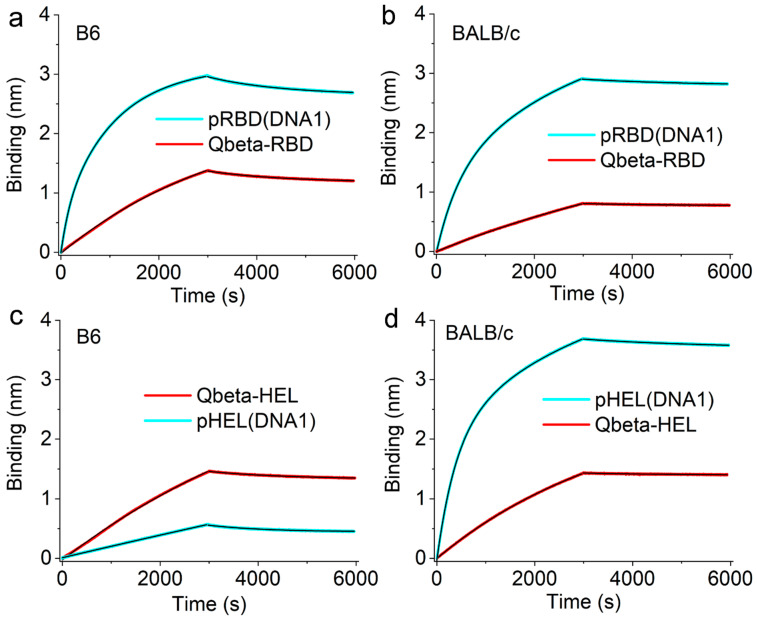
Side-by-side comparisons for real time binding and unbinding traces generated using animal sera collected on Day 123 after immunization with respective agents as shown in figure legends. (**a**,**b**) show traces from animals immunized with RBD-conjugated particulate Ags in B6 (**a**) and BALB/c mice (**b**), respectively. The doses of RBD were 0.24 µg per animal. (**c**,**d**) show traces from animals immunized with HEL-conjugated particulate Ags in B6 (**c**) and BALB/c mice (**d**), respectively. The doses of HEL on Qβ-HEL were 0.3 µg per animal, and the doses of HEL on pHEL(DNA1) were 0.1 µg per animal. All binding and unbinding traces can be well described by single-exponential kinetics, as shown by black curves overlaid on experimental data throughout this figure. Results are representative of at least two independent repeats of the same experiments.

**Figure 6:**
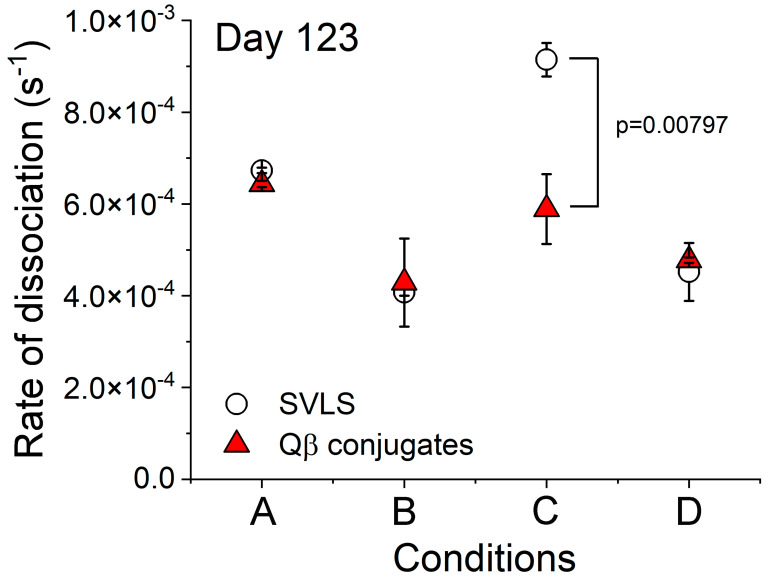
The rates of dissociation from respective targets for Ag-specific IgG in animal sera collected on Day 123 after animal immunization with respective particulate Ags. Condition A is for sera from B6 mice immunized with RBD-conjugated particulate Ags. Condition B is for sera from BALB/c mice immunized with RBD-conjugated particulate Ags. Condition C is for sera from B6 mice immunized with HEL-conjugated particulate Ags, and Condition D is for sera from BALB/c mice immunized with HEL-conjugated particulate Ags. All these rate constants were obtained from single-exponential fits to the unbinding traces directly measured from BioLayer interferometry (Figure 5). Immunization conditions for animals were the same as described in Figure 5. N = 4 (4 animals per group) for each data point.

Moreover, we have examined the Ag binding and unbinding traces for sera samples collected from animals on Day 123 after immunization with various agents. As shown in Figure 5, at the same sera dilution factors, all the traces from SVLS-immunized animals (cyan) showed much higher binding amplitudes than those animals immunized with Qβ-conjugates (red traces), except pHEL(DNA1) in B6 mice (Figure 5c). These results are consistent with ELISA data shown in Figure 2 and indicate higher Ag-specific IgG concentrations in SVLS-immunized animals. Furthermore, the unbinding traces allowed us to directly examine the off rates for IgG in binding to their respective Ags. As shown in Figure 6, between SVLS (circles) and Ags conjugated on Qβ VLPs (red triangles), the off rates for Ag-specific IgG in dissociation from their respective target were all identical within error, except for HEL-conjugated Ags in B6 mice. This is the case for Condition A in B6 mice between pRBD(DNA1) and Qβ-RBD, Condition B in BALB/c mice between pRBD(DNA1) and Qβ-RBD and Condition D in BALB/c mice between pHEL(DNA1) and Qβ- HEL. For Condition C, the off rate for HEL-specific IgG from B6 mice that were immunized with pHEL(DNA1) is 1.6-fold higher than that for HEL-specific IgG from B6 mice that were immunized with Qβ-HEL, with a *p*-value of 0.00797 from a one-way ANOVA test. This is fully consistent with the fact that pHEL(DNA1) was unable to induce affinity maturation in the B6 genetic background but the structural proteins in Qβ VLPs can recruit T cells to help accomplish affinity maturation, as shown in Figure 4c.

The data above on Ab affinity maturation and the comparison of Ab off rates from their respective targets demonstrate that both SVLS and Qβ platforms can induce comparable Ab affinity maturation in a suitable mouse host from a primary immune response, and these Abs differ little in their dissociation off rates from their target Ags. Yet the sera from mice immunized with Qβ-RBD had much lower potency in pseudovirion neutralization than the sera from mice immunized with pRBD(DNA1) (Figure 3). Altogether, these data suggest that the physical concentration of anti-RBD IgG induced by Qβ-RBD is much lower than that induced by SVLS in the same animal host.

To further test this, we have determined the physical concentration of Ag-specific IgG in animal sera using a method that is orthogonal to ELISA. This method works by capturing Ag-specific IgG on magnetic beads with increasing concentrations of Ag ‘bait’ to ensure complete capture of the Ag-specific Ab. We then quantify the amount of Ag-specific IgG captured using quantitative Western blotting, as we reported recently [21] (Figure S1). This approach does not have any assumption regarding affinities of the Ab and, thus, can yield the physical concentration for the Ag-specific IgG accurately.

**Figure 7:**
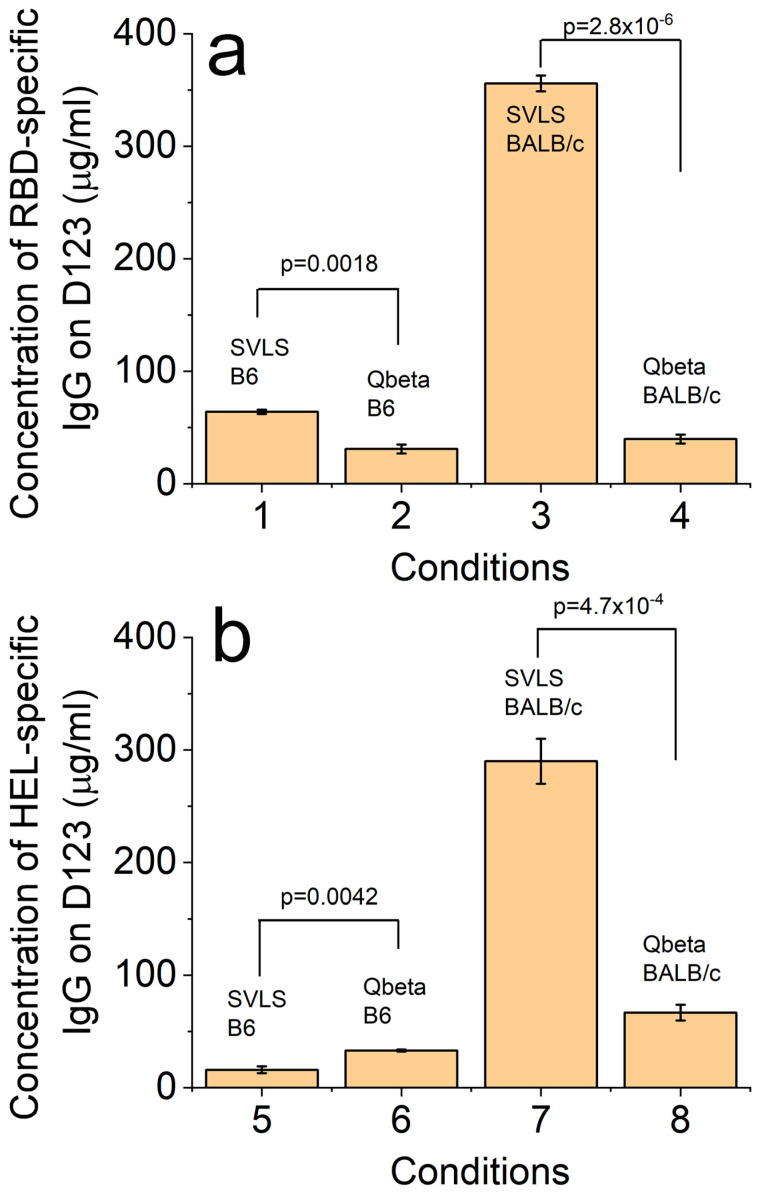
Concentrations of RBD-specific IgG (**a**) and HEL-specific IgG (**b**) in mouse sera collected on Day 123 post immunization with respective particulate Ags. These concentrations were measured by capture of the Ag-specific IgG on magnetic beads followed by quantitative Western blotting as described in Materials and Methods (Section 2.8). For Conditions 1 and 3, the SVLS was pRBD(DNA1). For Conditions 2 and 4, the Qβ was conjugated with RBD. For Conditions 5 and 7, the SVLS was pHEL(DNA1). For Conditions 6 and 8, the Qβ was conjugated with HEL.

Figure 7a shows the concentrations of RBD-specific IgG from mouse sera collected on Day 123 after immunization, which was the same time point as we used for the pseudovirion neutralization assay in Figure 3. First, most of these concentrations were higher than the concentrations reported by ELISA, as expected, because the concentration measured by ELISA depends on the affinity of the reference antibody. For any binding that is weaker than that between the reference antibody and the Ag, it will manifest as an apparent lower concentration due to more dissociation of the IgG during ELISA wash steps. Second, the concentration of anti-RBD IgG in the sera collected from B6 mice immunized with a single dose of pRBD(DNA1) (Condition 1) was 64 ± 2 µg/mL. This concentration is 2.1-fold higher than that of B6 mice after a single immunization of Qβ-RBD at the same Ag dose (Condition 2), with a *p*-value of 0.0018 from a one-way ANOVA test. Third, the concentration of anti-RBD IgG in the sera collected from BALB/c mice immunized with a single dose of pRBD(DNA1) (Condition 3) was 356 ± 7 µg/mL. This concentration is 8.9-fold higher than that of BALB/c mice after a single immunization of Qβ-RBD at the same Ag dose (Condition 4), with a *p*-value of 2.8 × 10^−6^ from a one-way ANOVA test. These results confirmed that the physical concentrations of anti-RBD IgG induced by Qβ-RBD were indeed much lower than those induced by SVLS in the same animal host.

Using the same technique of IgG capture followed by quantitative Western blotting, we also determined the concentrations of HEL-specific IgG from mouse sera collected on Day 123 after immunization with HEL-conjugated particulate Ags. As shown in Figure 7b, the concentration of anti-HEL IgG in the sera collected from B6 mice immunized with a single dose of pHEL(DNA1) (Condition 5) was 16 ± 3 µg/mL. This concentration is 2.1-fold less than that of B6 mice after a single immunization of Qβ-HEL (Condition 6), with a *p*-value of 0.0042 from a one-way ANOVA test, which was due to the lack of T cell help. In contrast, the concentration of anti-HEL IgG in the sera collected from BALB/c mice immunized with a single dose of pHEL(DNA1) (Condition 7) was 290 ± 20 µg/mL. This concentration is 4.3-fold higher than that of BALB/c mice after a single immunization of Qβ-HEL (Condition 8), with a *p*-value of 4.7 × 10^−4^ from a one-way ANOVA test. Therefore, within BALB/c mice, the SVLS platform is much more potent than the Qβ platform for the induction of Ag-specific IgG, as we have observed for both RBD and HEL.

**Figure 8:**
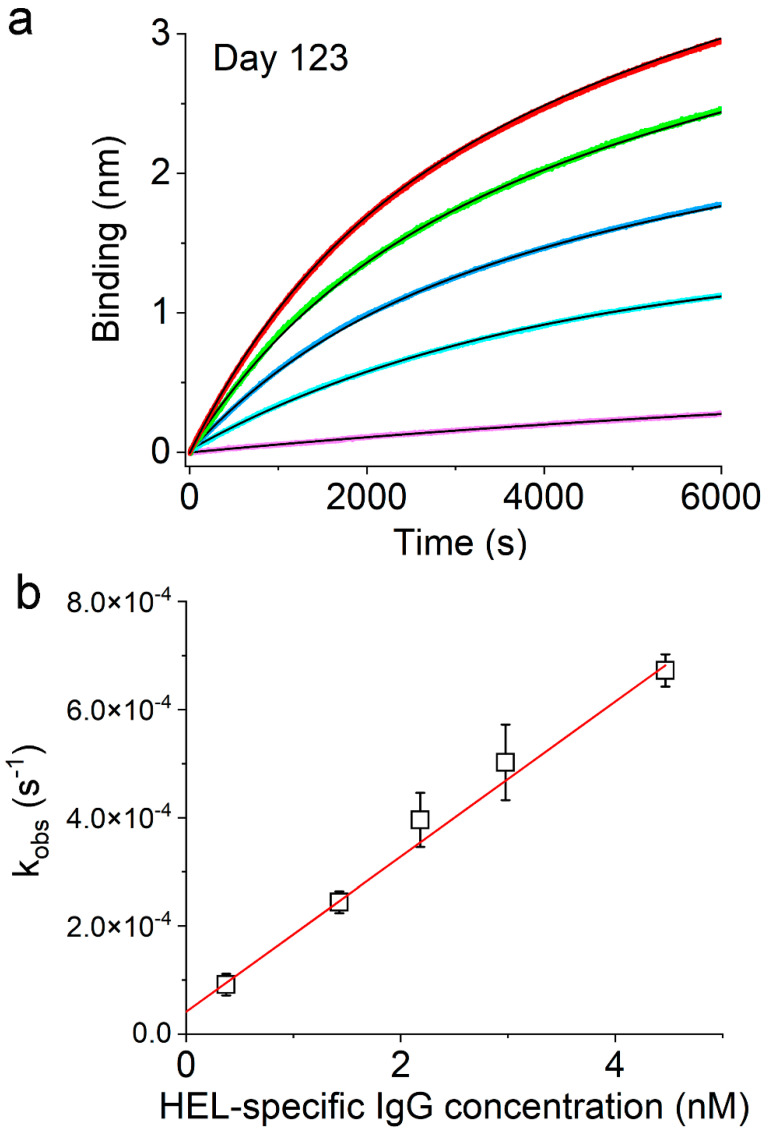
Measurement of the binding on-rate for HEL-specific IgG in the sera collected from BALB/c mice on Day 123 after a single immunization with Qβ-HEL at a dose of 0.3 µg per animal. (**a**) Real time binding curves from BioLayer interferometry experiments. The concentrations of HEL-specific IgG in each dilution, as represented by different colors in (**a**), are 0.37 nM (purple), 1.43 nM (cyan), 2.2 nM (blue), 3.0 nM (green), and 4.5 nM (red), which are plotted in panel (**b**) along the x-axis. The exponential fits are shown in black solid curves. (**b**) The observed binding rate constants with standard errors obtained from exponential fits of the binding curves shown in (**a**) are plotted as a function of HEL-specific IgG concentration. The adjusted R-square value from linear regressions is 0.995.

The availability of the physical concentration for the HEL-specific IgG allows us to determine the rate of IgG binding to HEL using the method that we reported recently [21]. This method entails BioLayer interferometry experiments at different concentrations of serum IgG to examine the dependence of the observed rate constants on IgG concentration (Section 2.7). Figure 8a shows the series of binding curves at different concentrations of the HEL-specific IgG, which were obtained from sera collected from BALB/c mice on Day 123 after a single immunization with Qβ-HEL. All of these binding curves can be well described by exponential functions (solid black lines). We plotted the observed rate constants from these exponential fits as a function of the HEL-specific IgG molar concentration in Figure 8b, which showed the expected linear dependence (squares) with an adjusted R-square value of 0.995 (straight red line). The slope of this line determined the bi-molecular rate constant for the binding between HEL and HEL-specific IgG, which was (1.43 ± 0.05) × 10^5^ M^−1^s^−1^ on Day 123. This rate constant is very close to that of HEL-specific IgG collected from BALB/c mice immunized with pHEL(DNA1), which was (1.29 ± 0.03) × 10^5^ M^−1^s^−1^ [21]. The equilibrium binding constant between HEL-specific IgG and HEL as determined from the on and off rate constants is, thus, (3.0 ± 0.4) × 10^8^ M^−1^ for BALB/c mice immunized with Qβ-HEL, which is identical within error to (2.9 ± 0.1) × 10^8^ M^−1^ for HEL-specific IgG from BALB/c mice immunized with pHEL(DNA1). These results are quantitatively consistent with the degree of Ab affinity maturation that we measured for these animals, where both Qβ-HEL and pHEL(DNA1) immunization triggered affinity maturation in BALB/c mice that were indistinguishable in I_am_ on Day 123 (Figure 4d). In conclusion, these HEL-specific IgGs had the same affinities for their target, but Qβ-HEL induced a much lower concentration of the Ab than SVLS in BALB/c mice.

## 5. Discussion

VLPs are a well-established form of vaccine [40,41], exemplified by the FDA-approved human papillomavirus vaccines with documented clinical safety and efficacy [1]. Despite the notable success of this platform in public health, much remains to be learned about the mechanisms that underlie the extraordinary immunogenicity of VLPs at the molecular and cellular levels [22]. Among various VLP platforms, bacteriophage Qβ VLPs have emerged as a model for understanding the immunogenicity of VLPs [5,10,11,12] and also proceeded to the stage of small-scale clinical trials for a potential hypertension vaccine [8]. To unravel the molecular basis of VLP immunogenicity, we have developed a reductionist system of synthetic virus-like structures (SVLS). These SVLS were constructed using highly purified biochemical components and engineered to resemble the common biochemical and biophysical features of naturally occurring viruses [15,16,25]. Compared to conventional VLPs such as Qβ that are produced directly from bacterial culture [9], the defined biochemical compositions of SVLS have allowed us to dissect the contributions of individual constituents to the overall immunogenicity of these structures [14,20,21]. Using Qβ as a well-established benchmark, we showed previously that SVLS rival Qβ-based VLPs in short-term Ab responses induced 11 days after immunization in mice [14]. Here, we have conducted this side-by-side comparison between SVLS and Qβ-based VLPs on a much longer time scale, up to one year after immunization. These data reveal that a single injection of either reagent in the absence of additional adjuvants can elicit long-lasting Ab responses in mice. However, SVLS induced much more potent Ab responses than Qβ-based VLPs in both B6 and BALB/c mice. In-depth biophysical characterization of these Ab responses revealed no differences in the affinities of the Abs induced by respective agents. Rather, it was the physical concentration of the Ag-specific Ab induced by Qβ-VLPs that was much lower than those induced by SVLS, suggesting that SVLS are more potent than Qβ-VLPs in the elicitation of long-lived plasma cells (LLPCs). LLPCs are cells that have been well recognized for their significance in vaccines because they can provide long-lasting protection against infection [42,43,44]. The formation, together with the characteristics of LLPCs after immunization or vaccination, has been a highly active topic for ongoing research [45,46]. Current data are consistent with two different scenarios: either more LLPCs were generated upon SVLS immunization, assuming all LLPCs had equal capacities in the secretion of Abs, or LLPCs with higher capabilities in the secretion of Abs were generated in response to SVLS immunization. Future studies are warranted to identify and characterize the LLPCs generated from these immunizations. On the other hand, the physical concentration of an Ab after immunization is highly significant for a prophylactic antiviral vaccine. It has been well demonstrated that above a minimum avidity threshold, protection in vivo simply depended on a minimum concentration of the Ab in the serum [47]. Therefore, a vaccine that can elicit high and durable concentrations of Ag-specific Abs is highly valued. Both the bacteriophage Qβ platform and the SVLS platform can achieve this, although SVLS accomplish this better in current studies. In this regard, SVLS may be a better alternative than Qβ-VLPs for the development of vaccines that can be further explored in the future, especially when a higher Ab concentration is desired for a long time.

In our current studies, we have not used any additional adjuvants for mouse immunization other than the molecular entities present in these two types of particulate Ags themselves. Therefore, the differences in immunization outcomes resulted from the differences in their compositions and supramolecular structures. From the perspective of immunogen compositions, SVLS only have the specific protein Ag, nonimmunogenic lipids, and CpG-containing DNA oligonucleotides that can activate TLR-9. The molecular compositions of Qβ-based VLPs are less well defined compared to SVLS, but they have the specific protein Ag, viral structural proteins, and single-stranded RNA, either from the Qβ viral genome or from *E. coli* cellular RNA that will activate TLR-7. Based on these differences, it is notable that the presence of additional viral structural proteins in Qβ-based VLPs does not offer a substantial advantage to the vaccine formulation when the protein Ag of interest is able to elicit T cell help by itself, exemplified by HEL in BALB/c mice (Figure 2d). In fact, this is also the case for RBD in B6 mice, for which we have data (Wholey & Cheng, unpublished) to indicate that RBD by itself can recruit T cell help in B6 mice. As shown in Figure 2a, pRBD(DNA1) induced much higher anti-RBD IgG than Qβ-RBD, even though Qβ structural proteins can provide additional T cell help for this response. This T cell help elicited by RBD also distinguishes RBD from HEL in the B6 background, in which pHEL(DNA1) can only induce limited T cell independent anti-HEL IgG responses in B6 mice (Figure 2c stars). It remains to be determined how the RBD protein elicits T cell help in B6 mice and whether it relates to the glycosylation of RBD. In summary, while it is tempting to speculate that the CpG DNA inside SVLS may be a key driver for the potent and durable Ab responses, especially in light of our recent studies [14,21], how T cell help and different TLR activation coordinate to result in balanced germinal center and plasma cell responses remains a subject of future research.

Lastly, from the perspective of immunogen structures, there is one major difference between SVLS and Qβ-VLPs. While SVLS are lipid bilayer-based structures that share similarities with enveloped viruses, Qβ is an icosahedral virus, and it is known that the structural and mechanical features of icosahedral viruses are different from those of enveloped viruses [2]. Rigidity and fluidity of the structures [48] are both likely to influence Ab responses [49]. In relation to the fluidity of the structures, a very important parameter for B cell antibody responses is epitope spacing [50,51]. While the epitope spacing is variable in real time on SVLS due to the lateral diffusion of lipid molecules within the lipid bilayer [52,53], the epitope spacing on Qβ-VLPs is relatively fixed due to the rigid icosahedral structures of the particles. In the current study, we conjugate proteins onto the surface of SVLS or Qβ-VLPs, while for many VLPs derived from enveloped viruses, viral surface Ags are integral membrane proteins that have membrane-spanning domains and cytoplastic domains. The flexibility of these proteins in their lateral diffusion within the plane of the viral membrane is likely to be very different from those proteins conjugated on the SVLS surface (Figure 1). Mechanistically, how these different biophysical features of a particulate Ag contribute to Ab responses remains to be investigated in the future. Understanding of the immunogenicity of SVLS will also benefit from more structural studies on these potent immunogens.

## 6. Conclusions

Using the mouse as our model system, we show that synthetic virus-like structures (SVLS), a new platform that we have developed recently to understand the molecular basis of viral immunogenicity, can induce much more potent neutralizing antibody responses than the well-established Qβ-based virus-like particles (VLPs). Thus, SVLS may be a better alternative than Qβ-VLPs for the development of vaccines that can be explored in the future.

## Data Availability

Data are contained within the article.

## Supplementary Materials

The following supporting information can be downloaded at: https://www.mdpi.com/article/10.3390/v17040579/s1. Figure S1: IgG capture followed by quantitative Western blotting to measure the concentration of Ag-specific IgG in mouse sera.

## Abbreviations

The following abbreviations are used in this manuscript: SVLS, synthetic virus-like structures; VLPs, virus-like particles; Ag, antigen; Ab, antibody; TLR, Toll-like receptor; ED, epitope density; RBD, receptor binding domain of SARS-CoV-2; HEL, hen egg lysozyme; sRBD, soluble RBD; sHEL, soluble HEL; pRBD(DNA1), particulate antigen with RBD covalently conjugated on SVLS surface, with DNA1 encapsulated in the interior of SVLS; pHEL(DNA1), particulate antigen with HEL covalently conjugated on SVLS surface, with DNA1 encapsulated in the interior of SVLS; Qβ-RBD, particulate antigen with RBD covalently conjugated on Qβ-VLP surface; Qβ-HEL, particulate antigen with HEL covalently conjugated on Qβ-VLP surface; I_am_, indicator of affinity maturation; LLPCs, long-lived plasma cells.

## References

[1] Schiller J.T., Lowy D.R. (2011). Developmental History of HPV Prophylactic Vaccines. History of Vaccine Development.

[2] Knipe D.M., Howley P.M. (2013). Fields Virology.

[3] Bachmann M.F., Rohrer U.H., Kundig T.M., Burki K., Hengartner H., Zinkernagel R.M. (1993). The influence of antigen organization on B cell responsiveness. Science.

[4] Chackerian B., Lenz P., Lowy D.R., Schiller J.T. (2002). Determinants of autoantibody induction by conjugated papillomavirus virus-like particles. J. Immunol..

[5] Chackerian B., Durfee M.R., Schiller J.T. (2008). Virus-like display of a neo-self antigen reverses B cell anergy in a B cell receptor transgenic mouse model. J. Immunol..

[6] Jegerlehner A., Storni T., Lipowsky G., Schmid M., Pumpens P., Bachmann M.F. (2002). Regulation of IgG antibody responses by epitope density and CD21-mediated costimulation. Eur. J. Immunol..

[7] Kozlovska T.M., Cielens I., Dreilinna D., Dislers A., Baumanis V., Ose V., Pumpens P. (1993). Recombinant RNA phage Q beta capsid particles synthesized and self-assembled in Escherichia coli. Gene.

[8] Tissot A.C., Maurer P., Nussberger J., Sabat R., Pfister T., Ignatenko S., Volk H.D., Stocker H., Muller P., Jennings G.T. (2008). Effect of immunisation against angiotensin II with CYT006-AngQb on ambulatory blood pressure: A double-blind, randomised, placebo-controlled phase IIa study. Lancet.

[9] Van Rompay K.K., Hunter Z., Jayashankar K., Peabody J., Montefiori D., LaBranche C.C., Keele B.F., Jensen K., Abel K., Chackerian B. (2014). A vaccine against CCR5 protects a subset of macaques upon intravaginal challenge with simian immunodeficiency virus SIVmac251. J. Virol..

[10] Hou B., Saudan P., Ott G., Wheeler M.L., Ji M., Kuzmich L., Lee L.M., Coffman R.L., Bachmann M.F., DeFranco A.L. (2011). Selective utilization of Toll-like receptor and MyD88 signaling in B cells for enhancement of the antiviral germinal center response. Immunity.

[11] Jegerlehner A., Maurer P., Bessa J., Hinton H.J., Kopf M., Bachmann M.F. (2007). TLR9 signaling in B cells determines class switch recombination to IgG2a. J. Immunol..

[12] Hong S., Zhang Z., Liu H., Tian M., Zhu X., Zhang Z., Wang W., Zhou X., Zhang F., Ge Q. (2018). B Cells Are the Dominant Antigen-Presenting Cells that Activate Naive CD4(+) T Cells upon Immunization with a Virus-Derived Nanoparticle Antigen. Immunity.

[13] Chang J.Y., Gorzelnik K.V., Thongchol J., Zhang J. (2022). Structural Assembly of Qbeta Virion and Its Diverse Forms of Virus-like Particles. Viruses.

[14] Wholey W.-Y., Meyer A.R., Yoda S.-T., Mueller J.L., Mathenge R., Chackerian B., Zikherman J., Cheng W. (2024). An integrated signaling threshold initiates IgG response towards virus-like immunogens. J. Immunol..

[15] Wholey W.-Y., Mueller J.L., Tan C., Brooks J.F., Zikherman J., Cheng W. (2020). Synthetic Liposomal Mimics of Biological Viruses for the Study of Immune Responses to Infection and Vaccination. Bioconjug. Chem..

[16] Wholey W.-Y., Yoda S.T., Cheng W. (2021). Site-Specific and Stable Conjugation of the SARS-CoV-2 Receptor-Binding Domain to Liposomes in the Absence of Any Other Adjuvants Elicits Potent Neutralizing Antibodies in BALB/c Mice. Bioconjug. Chem..

[17] Harris A., Cardone G., Winkler D.C., Heymann J.B., Brecher M., White J.M., Steven A.C. (2006). Influenza virus pleiomorphy characterized by cryoelectron tomography. Proc. Natl. Acad. Sci. USA.

[18] Briggs J.A., Wilk T., Welker R., Krausslich H.G., Fuller S.D. (2003). Structural organization of authentic, mature HIV-1 virions and cores. EMBO J..

[19] Ke Z., Oton J., Qu K., Cortese M., Zila V., McKeane L., Nakane T., Zivanov J., Neufeldt C.J., Cerikan B. (2020). Structures and distributions of SARS-CoV-2 spike proteins on intact virions. Nature.

[20] Brooks J.F., Riggs J., Mueller J.L., Mathenge R., Wholey W.-Y., Meyer A.R., Yoda S.T., Vykunta V.S., Nielsen H.V., Cheng W. (2023). Molecular basis for potent B cell responses to antigen displayed on particles of viral size. Nat. Immunol..

[21] Wholey W.-Y., Meyer A.R., Yoda S.T., Chackerian B., Zikherman J., Cheng W. (2024). Minimal determinants for lifelong antiviral antibody responses from a single exposure to virus-like immunogens at low doses. Vaccines.

[22] Cheng W. (2016). The Density Code for the Development of a Vaccine?. J. Pharm. Sci..

[23] Klein J.S., Bjorkman P.J. (2010). Few and far between: How HIV may be evading antibody avidity. PLoS Pathog..

[24] Chen Z., Moon J.J., Cheng W. (2018). Quantitation and Stability of Protein Conjugation on Liposomes for Controlled Density of Surface Epitopes. Bioconjug. Chem..

[25] Chen Z., Wholey W.-Y., Hassani Najafabadi A., Moon J.J., Grigorova I., Chackerian B., Cheng W. (2020). Self-Antigens Displayed on Liposomal Nanoparticles above a Threshold of Epitope Density Elicit Class-Switched Autoreactive Antibodies Independent of T Cell Help. J. Immunol..

[26] Pang Y., Song H., Kim J.H., Hou X., Cheng W. (2014). Optical trapping of individual human immunodeficiency viruses in culture fluid reveals heterogeneity with single-molecule resolution. Nat. Nanotechnol..

[27] DeSantis M.C., Kim J.H., Song H., Klasse P.J., Cheng W. (2016). Quantitative Correlation between Infectivity and Gp120 Density on HIV-1 Virions Revealed by Optical Trapping Virometry. J. Biol. Chem..

[28] Cui Z., Gorzelnik K.V., Chang J.Y., Langlais C., Jakana J., Young R., Zhang J. (2017). Structures of Qbeta virions, virus-like particles, and the Qbeta-MurA complex reveal internal coat proteins and the mechanism of host lysis. Proc. Natl. Acad. Sci. USA.

[29] Lan J., Ge J., Yu J., Shan S., Zhou H., Fan S., Zhang Q., Shi X., Wang Q., Zhang L. (2020). Structure of the SARS-CoV-2 spike receptor-binding domain bound to the ACE2 receptor. Nature.

[30] Kondo H., Shiroishi M., Matsushima M., Tsumoto K., Kumagai I. (1999). Crystal structure of anti-Hen egg white lysozyme antibody (HyHEL-10) Fv-antigen complex. Local structural changes in the protein antigen and water-mediated interactions of Fv-antigen and light chain-heavy chain interfaces. J. Biol. Chem..

[31] Connor R.I., Chen B.K., Choe S., Landau N.R. (1995). Vpr is required for efficient replication of human immunodeficiency virus type-1 in mononuclear phagocytes. Virology.

[32] England C.G., Ehlerding E.B., Cai W. (2016). NanoLuc: A Small Luciferase Is Brightening Up the Field of Bioluminescence. Bioconjug. Chem..

[33] Yurkovetskiy L., Wang X., Pascal K.E., Tomkins-Tinch C., Nyalile T.P., Wang Y., Baum A., Diehl W.E., Dauphin A., Carbone C. (2020). Structural and Functional Analysis of the D614G SARS-CoV-2 Spike Protein Variant. Cell.

[34] Kim J.H., Song H., Austin J.L., Cheng W. (2013). Optimized Infectivity of the Cell-Free Single-Cycle Human Immunodeficiency Viruses Type 1 (HIV-1) and Its Restriction by Host Cells. PLoS ONE.

[35] Yasmeen A., Ringe R., Derking R., Cupo A., Julien J.P., Burton D.R., Ward A.B., Wilson I.A., Sanders R.W., Moore J.P. (2014). Differential binding of neutralizing and non-neutralizing antibodies to native-like soluble HIV-1 Env trimers, uncleaved Env proteins, and monomeric subunits. Retrovirology.

[36] Zhou P., Yang X.L., Wang X.G., Hu B., Zhang L., Zhang W., Si H.R., Zhu Y., Li B., Huang C.L. (2020). A pneumonia outbreak associated with a new coronavirus of probable bat origin. Nature.

[37] Sercarz E.E., Lehmann P.V., Ametani A., Benichou G., Miller A., Moudgil K. (1993). Dominance and crypticity of T cell antigenic determinants. Annu. Rev. Immunol..

[38] Grewal I.S., Moudgil K.D., Sercarz E.E. (1995). Hindrance of binding to class II major histocompatibility complex molecules by a single amino acid residue contiguous to a determinant leads to crypticity of the determinant as well as lack of response to the protein antigen. Proc. Natl. Acad. Sci. USA.

[39] Schmidt F., Weisblum Y., Muecksch F., Hoffmann H.H., Michailidis E., Lorenzi J.C.C., Mendoza P., Rutkowska M., Bednarski E., Gaebler C. (2020). Measuring SARS-CoV-2 neutralizing antibody activity using pseudotyped and chimeric viruses. J. Exp. Med..

[40] Bachmann M.F., Jennings G.T. (2010). Vaccine delivery: A matter of size, geometry, kinetics and molecular patterns. Nat. Rev. Immunol..

[41] Chackerian B., Lowy D.R., Schiller J.T. (2001). Conjugation of a self-antigen to papillomavirus-like particles allows for efficient induction of protective autoantibodies. J. Clin. Investig..

[42] Manz R.A., Thiel A., Radbruch A. (1997). Lifetime of plasma cells in the bone marrow. Nature.

[43] Slifka M.K., Antia R., Whitmire J.K., Ahmed R. (1998). Humoral immunity due to long-lived plasma cells. Immunity.

[44] Chackerian B., Peabody D.S. (2020). Factors That Govern the Induction of Long-Lived Antibody Responses. Viruses.

[45] Nutt S.L., Hodgkin P.D., Tarlinton D.M., Corcoran L.M. (2015). The generation of antibody-secreting plasma cells. Nat. Rev. Immunol..

[46] Ripperger T.J., Bhattacharya D. (2021). Transcriptional and Metabolic Control of Memory B Cells and Plasma Cells. Annu. Rev. Immunol..

[47] Bachmann M.F., Kalinke U., Althage A., Freer G., Burkhart C., Roost H., Aguet M., Hengartner H., Zinkernagel R.M. (1997). The role of antibody concentration and avidity in antiviral protection. Science.

[48] Marchetti M., Wuite G., Roos W.H. (2016). Atomic force microscopy observation and characterization of single virions and virus-like particles by nano-indentation. Curr. Opin. Virol..

[49] Watson D.S., Endsley A.N., Huang L. (2012). Design considerations for liposomal vaccines: Influence of formulation parameters on antibody and cell-mediated immune responses to liposome associated antigens. Vaccine.

[50] Dintzis H.M., Dintzis R.Z., Vogelstein B. (1976). Molecular determinants of immunogenicity: The immunon model of immune response. Proc. Natl. Acad. Sci. USA.

[51] Veneziano R., Moyer T.J., Stone M.B., Wamhoff E.C., Read B.J., Mukherjee S., Shepherd T.R., Das J., Schief W.R., Irvine D.J. (2020). Role of nanoscale antigen organization on B-cell activation probed using DNA origami. Nat. Nanotechnol..

[52] Jacobson K. (1983). Lateral diffusion in membranes. Cell Motil..

[53] Lee G.M., Zhang F., Ishihara A., McNeil C.L., Jacobson K.A. (1993). Unconfined lateral diffusion and an estimate of pericellular matrix viscosity revealed by measuring the mobility of gold-tagged lipids. J. Cell Biol..

