# Supplementary material for: Superior Potency of Synthetic Virus-like Structures in Vaccine-Induced Antibody Responses Compared to Qβ Bacteriophage Virus-like Particles
Source: Viruses. 2025 Apr 17;17(4):579. doi: 10.3390/v17040579 (PMC12030905; doi:10.3390/v17040579)
Supplement: Supplementary file 1 [file viruses-17-00579-s001.zip › viruses-3546482-supplementary.pdf]

Supporting Information for

Superior potency of synthetic virus-like structures in vaccine-induced antibody responses compared to Qβ bacteriophage virus-like particles

Alexander R. Meyer[1†], Libo Li[1†], Wei-Yun Wholey[1], Bryce Chackerian[2], Wei Cheng[1,3]*

[1]*Department of Pharmaceutical Sciences, 428 Church Street, University of Michigan, Ann Arbor, Michigan 48109, USA*

[2]*Department of Molecular Genetics and Microbiology, School of Medicine, University of New Mexico, Albuquerque, New Mexico 87131, USA*

[3]*Department of Biological Chemistry, 1150 W. Medical Center Dr., University of Michigan Medical School, Ann Arbor, Michigan 48109, USA*

[†]Both authors contributed equally to this work.

*Correspondence to: chengwe@umich.edu

The supporting information includes:

Supporting Figure S1

Figure S1. IgG capture followed by quantitative Western blotting to measure the concentration of Ag-specific IgG in mouse sera.

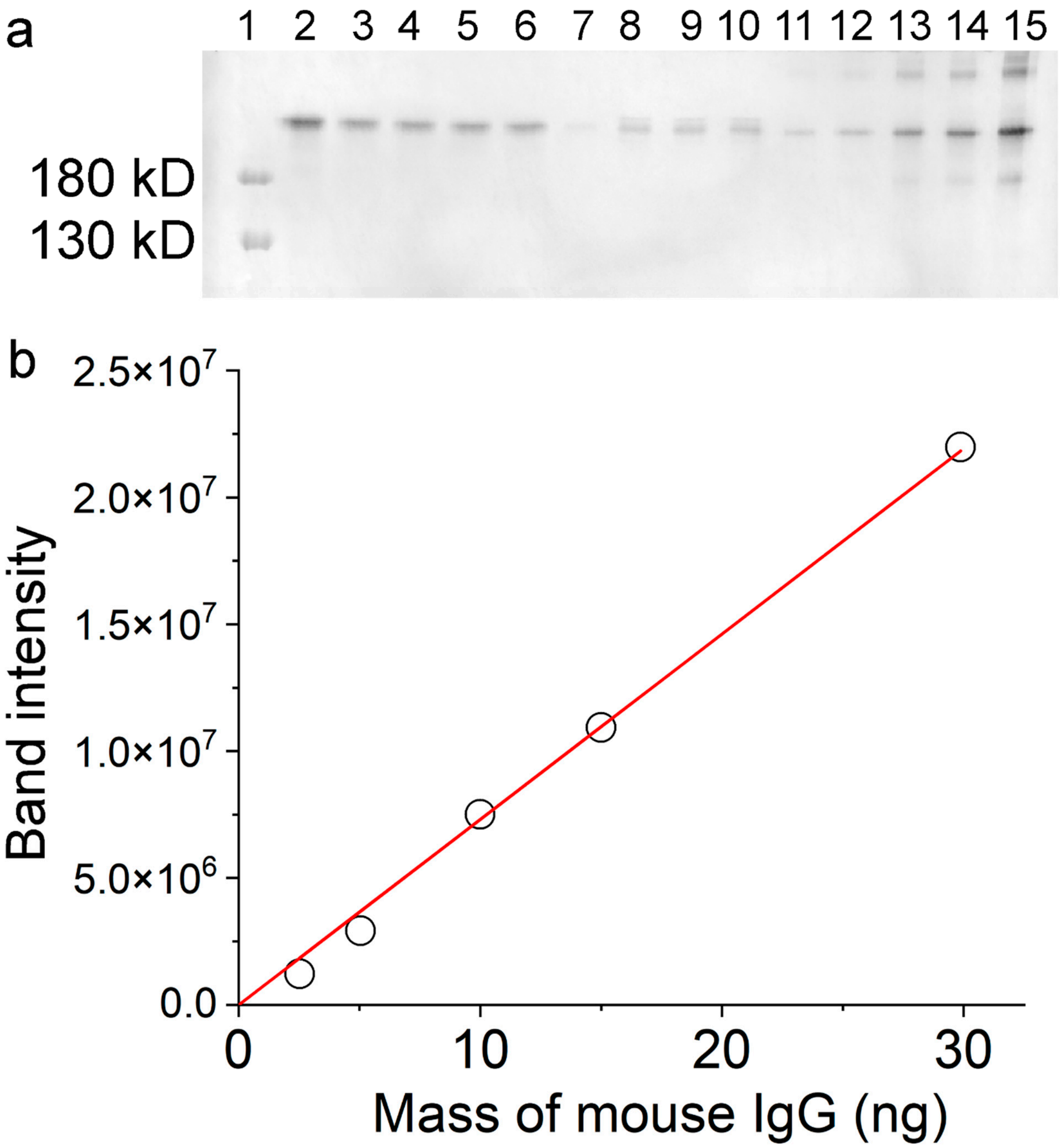

(a) A representative example of a Western blotting image to illustrate our method of Ag-specific IgG quantitation in mouse sera. From left to right, the lanes numbered 1 through 15 are: prestained marker, initial serum sample before magnetic bead capture, supernatant from capture control (no biotinylated RBD was on the magnetic beads), supernatants from capture with increasing concentrations of biotinylated RBD on the magnetic beads (lanes 4, 5, and 6), captured control sample (no biotinylated RBD was on the magnetic beads), captured IgG with increasing concentrations of biotinylated RBD on the magnetic beads (lanes 8, 9, and 10), 2.53,

5.05, 10, 15 and 29.87 ng reference mouse IgG (mAb1, a commercial purified monoclonal mouse IgG, BioLegend CAT#944803). (b) Band intensities from lanes 11 through 15 were used as the known reference to construct a standard curve to quantify the RBD-specific IgG antibodies captured by streptavidin magnetic beads. The band intensity was quantified using Image Lab version 6.1 (Bio-Rad) and plotted against the loaded references. All visible bands were included for standard curve construction. A linear regression (the straight red line) was then used to quantitate the mass of Ag-specific IgG in serum samples. The adjusted R-square value from the linear regression as shown above is 0.998. The mass was then used to calculate the concentration of the IgG based on volume dilutions.
